# Cognitive-Behavioural therapy and interpersonal psychotherapy for the treatment of post-natal depression: a narrative review

**DOI:** 10.1186/s40359-018-0240-5

**Published:** 2018-06-18

**Authors:** George Stamou, Azucena García-Palacios, Cristina Botella

**Affiliations:** 1 Brief Intervention Service, WellSouth, 333 Princes Street, Dunedin, New Zealand; 2 Universitat Jaume I, Castellón, Spain; 3 Department of Basic and Clinical Psychology and Psychobiology, Universtitat Jaume I, Department of Psychology, Castellón, Spain

**Keywords:** Post-natal depression, Psychological treatments, Virtual reality, Narrative review

## Abstract

**Background:**

Post-natal Depression (PND) is a depressive disorder that causes significant distress or impairment on different levels in the individual’s life and their families. There is already evidence of the efficacy of psychological treatments for PND. We conducted a narrative review and researched the literature for identifying systematic reviews and studies for the best psychological treatments of PND, and examined what parameters made those treatments successful.

**Methods:**

We searched 4 electronic databases. We included reviews and randomised controlled clinical trials for our research. We excluded other types of studies such as case studies and cohort studies.

We followed a specific search strategy with specific terms and a selection process. We identified risk of bias in reviews and studies, and identified their limitations. We synthesized the data based on particular information, including: name of the authors, location, research type, target, population, delivery, outcome measures, participants, control groups, types of intervention, components of treatments, providers, experimental conditions amongst others.

**Results:**

We found 6 reviews and 15 studies which met our inclusion criteria focusing on Cognitive Behavioural Therapy (CBT) for PND.

Among the main findings we found that CBT can be delivered on an individual basis or within a group. It can be effective in the short-term, or up to six months post-intervention. CBT can be delivered by professionals or experts, but can also be practiced by non-experts.

We found 7 components of CBT, including psychoeducation, cognitive restructuring, and goal setting.

We also researched whether virtual reality (VR) has ever been used for the treatment of PND, and found that it has not.

**Conclusion:**

From our review, we have concluded that CBT is an effective treatment for PND. We have explored the utility of VR as a possible therapeutic modality for PND and have decided to run a pilot feasibility study as a next step, which will act as the foundational guide for a clinical trial at a later stage.

## Background

### Post-natal depression: Definition, clinical features, risk factors, and effects

Post-partum period has been associated with mood disturbances since Hippocrates’ times [1]. It is reported that PND started being officially used in psychiatric manuals in 1994 [2]. It was “officially objectified” in the 1950s [3]. PND was given some “diagnostic criteria” at that time as practitioners began to notice that some women, after giving birth, would experience a psychological pattern with depressive characteristics.

In the earlier days, science was trying to understand PND in the context of causes. These varied from the socio-economic background of the individual to unplanned pregnancy [4]. In more recent years, clinicians have gained a better understanding of PND. They give emphasis to the combination of risk factors rather than single causes [5]. Risk factors can vary from low socio-economic background, family history of depression or personal history of mental health issues, low social support, smoking habits, sexuality issues, and immigration issues [6].

The Diagnostic and Statistical Manual of Mental Disorders - fourth edition (DSM-IV) [7] initially linked PND with major depression with post-partum onset within 4 weeks of birth. However, a study for the treatment of PND [8] highlights the variations amongst studies which define the onset of PND, from the first month up to the end of the first year following the infant’s birth.

The Diagnostic and Statistical Manual of Mental Disorders, DSM-5 [9] places PND in the category of unspecified depressive disorders where the main symptoms can cause significant distress or impairment on various levels in the individual’s life. PND includes different sub-categories called specifiers, amongst which are the peri-partum onset. This refers to the onset of depression during pregnancy or postpartum for the time following the birth of the child. According to the same manual, a large number of postpartum major depressive episodes begin during pregnancy, thus they are also called peri-partum episodes. These episodes range from mild to severe, with or without psychotic features. The individual may also experience hallucinations or delusions.

A study by Hewitt et al. [10] describes depression as a very serious mental health problem with important consequences on a societal level. In this study they estimate that depression will become the second-highest health problem by 2020. According to the same authors, PND is considered to be a very important category of depression with often serious consequences. It can affect both the mother and the infant, as well as the immediate and/or the extended family. PND can have long-lasting effects on the development of the infant on a cognitive and emotional level, including attachment issues amongst others [11].

PND can cause significant distress or impairment on various levels in the individual’s life, e.g. lack of motivation, affected mood, sleep and appetite issues, lack of concentration, rumination, unintentional or intentional suicidal ideation, or psychotic phenomena such as hallucinations or delusions.

PND affects 13% of women [12], and between 4 and 25% in men in the first 2 months after the baby is born [13]. Other research suggests that the proportion of mothers who suffer from PND is one in seven [14]. According to the same article, the mentality of organisations and health providers is changing, especially in the United States where there is a shift towards more systematic screening of mothers-to-be or young mothers who might experience symptoms of depression.

### Psychological treatments of PND

Regarding treatment for PND, Rudlin lists its main therapeutic approaches [15]. They vary from medication, home visits, education, phone contact, one-to-one counselling, group therapy, and self-help resources such as books. CBT, together with interpersonal psychotherapy (IPT) are considered two efficacious non-pharmacological treatments for PND [16].

A meta-analysis [17] examined how effective psychological treatments are for PND in primary care. It was found that psychological interventions such as CBT and IPT, along with counselling, psychodynamic therapy and support groups can be very effective in reducing the symptoms of depression up to 6 months post-intervention.

CBT’s main focus is identifying distorted negative thinking patterns. It emphasises the link between thoughts, feelings and behaviour. Dalby [18] highlights Albert Ellis’s theory of irrational thinking patterns and how they could trigger emotional disturbance to the individual.

A common characteristic of people who suffer from depression is their tendency to experience automatic thoughts, usually of negative content. Beck [19] highlights that the negative automatic thoughts usually carry negative meaning in relation to the notion of the past or the future, about the individual themselves, and/or the world around the person.

CBT helps the individual to understand that identifying their own distorted negative thinking patterns allows them an opportunity to change them. By changing their thinking, the individual can change how they view and feel about themselves, and ultimately, change their behaviour [20]. CBT integrates many approaches in clinical practice such as problem solving, modelling, and cognitive restructuring, amongst others [21].

IPT focuses on four areas in the person’s life: grief about someone’s own sense of self or changes within their relationships, changes in roles, unresolved disagreements in interpersonal relationships, and a lack of life events. It focuses on strengthening the relationships of the individual, on increasing social support, and improves communication [22].

Conversely, Barlow [23] refers to the negative effects, or no effects of psychological interventions for various disorders, including trauma and addictions. Dimidjian and Hollon [24] talk about the adverse effect of psychotherapy, but this is yet to be researched adequately. There is no real agreement in the scientific community about ways to investigate and identify harmful psychological interventions. A review [25] published by the World Health Organisation (WHO) for psychological interventions on depression discusses the under-investigated but very real possibility of a negative effect of psychological therapies on depression. Some of those effects include the symptomatology of the individual becoming exacerbated or the individual experiencing a “relapse”.

Lambert [26] makes the point that psychological treatments for depression and other disorders have shown to be effective overall. Positive outcomes depend on the patients’ characteristics, but also the therapists’ “actions” or “inactions”. Lambert also highlights the reality of negative clinical outcomes for patients who experience depression. Lambert does, however, identify ways to minimise clinical negative impact and maximise positive outcomes which can be achieved through “measuring, monitoring, and tracking client treatment response with standardised scales”.

A meta-analysis by Cuijpers et al. [27], which we did not include in our initial search, investigates the effect of psychological treatments for PND. It was found that CBT, IPT, counselling and social support have an overall positive effect on PND, but they were less effective than what they have been on other psychological disorders. There was no real difference in therapeutic outcome between different psychological therapies. The same study also concluded that medication and electroconvulsive therapy can have higher effect size for PND than psychological treatments but that needs to be further investigated. In addition, it was found that the initial positive therapeutic effect of psychological treatments on PND could not be confirmed 6months or longer post-intervention. However, the authors of this meta-analysis highlight that some of these findings need to be interpreted with caution due to the small number of studies included, and that the quality of the studies was not the highest.

### Virtual reality: Definition and its advantages

A question worthy of investigating in relation to the treatments of PND is whether they can be improved for better clinical outcomes using other treatments, such as virtual reality (VR).

VR is defined as “a way for humans to visualise, manipulate, and interact with computers, and it can be viewed as an advanced form of human-computer interface that allows the user to interact immersed in more intuitive and naturalistic fashion” [28].

VR promotes a sense of presence for the user in an environment which is computer based. According to Turner and Casey [29], VR can enhance the therapeutic effectiveness of psychological interventions. It expands beyond the strict boundaries of technology. VR is seen as a form of communication. It comprises elements such as an experience, visualisation and interaction [30].

Some of VR’s advantages in research and practice is that it can act as a powerful and effective tool which can complement traditional therapies such as CBT [31]. It is a form of therapy which enhances sense of control and raises self-efficacy. It uses technological means to help the individual. It is 3-dimensional and interactive. The VR user has the ability to “explore and engage” within the virtual environment.

VR can be delivered in a safe and controlled way [28]. It can be affordable, easy to access, and the therapist themselves can have control of how, what and when it is to be applied, which creates a sense of safety for the user [32]. One of VR’s main features and characteristics is that it can empower the individual, a very basic and essential ingredient in order for therapy to occur. The combination of CBT with VR can have a tremendous positive impact [29].

VR or virtual reality exposure therapy (VRET) has been used to explore a large number of topics, from stress, anxiety, phobias, acute pain, body image disturbances, eating disorders, training of children in spatial and navigation learning skills, functional skills [28], post-traumatic stress disorder (PTSD) [33–35], fear of heights and also fear of flying [36, 37].

### Improvement of traditional treatments for PND

There appears to be a gap in the literature relating to the combination of VR with traditional therapies for the treatment of PND. A brief literature review so far identified only one study on the efficacy of VR on depression. A pilot study conducted by Falconer et al. investigated the concept of compassion and self-criticism in a virtual environment [38]. They examined whether compassion could be taught to subjects in a virtual environment. The study revealed that its participants, all adults with depression, were able to practice compassion both as a life-sized avatar and as a child avatar interacting with one another through the process of embodiment. Although this particular study was limited, being a small group (*n* = 15), and having no control group, its results were promising. Results indicated that most of the study’s participants had become more compassionate and less self-critical one-month post-intervention.

Our research group is exploring the utility of using VR in the treatment of PND, the final aim being to improve the treatment that mothers with PND receive. This could potentially be beneficial for the health and well-being of mothers, their families, and society in general. The investigation of the combination of CBT and VR could possibly provide a better treatment for PND from a clinical point of view, which could save on resources including time and money spent at an organisational level.

In order to explore the possibility of combining psychological therapies with VR for the treatment of PND, we decided to review past and current published literature on traditional therapies for PND. We wanted to find out what therapies work best, and under what circumstances. Our investigation researched other reviews, within the same clinical subject area. However, our review differs from other reviews, whether systematic or narrative reviews, in three methodological aspects. It focuses purely on the treatment of PND, rather than prevention, or prevention and treatment of PND. A second difference is that this review investigates mainly CBT as treatment for PND. A third advantage of this review is around population characteristics. We focused our research mainly on the post-partum clinical population and not on other types such as the ante-natal population. However, there was one exception where the clinical population was in the last trimester of their pregnancy in the beginning of the study, but it became post-partum at a later stage. We believe this three research characteristics help this research project make a clear contribution to the literature.

### Objectives

We formulated the design of this review based on the working hypothesis that CBT is a successful treatment for various psychological disorders, amongst them PND. It is a therapeutic approach which is scientific based. It can follow a clinical protocol, where its clinical methods can be replicated. Its clinical efficacy can be tested and measured. We hypothesised that CBT is the most widely used and efficacious treatment for depression and PND.

We searched for specific parameters which we believe contribute to the efficacy of CBT. We wanted to pay particular attention to the types of participants, especially the ones who had been diagnosed with PND through a structured clinical interview. Another parameter was around the types of interventions or treatment components of CBT for PND. We searched for specific aspects of the CBT approach, in particular, cognitive restructuring, goal setting, and problem-solving.

In order to start this line of research and to design our PND intervention protocol, supported with VR, our first aim is to review the scientific literature relating to the most effective CBT treatments for PND. Then to identify the parameters that make those treatments effective. It will also investigate whether VR has previously been used as a treatment for PND.

More specifically, this review will answer the following three research questions:

1. What CBT psychological treatments are effective for PND?
2. What are the parameters that make those treatments have a successful clinical outcome?
3. Has VR previously been used for the treatment of PND?

## Methods

Studies for this review were selected according to specific criteria. The studies which we included for this review were reviews and randomised controlled trials. Case studies, cohort studies, or cluster trials were excluded. The reason for including randomised controlled trials and excluding other types of studies, such as case studies, was that randomised controlled trials are considered to be the “gold standard of clinical trials” [39].

We included studies which investigated the treatment of PND. We excluded studies that investigated the prevention of PND or treatment of post-natal anxiety. We included studies where treatments were delivered in home based or in public settings such as clinics or hospitals.

There were no restrictions around the intervention providers in the included studies. They varied from professionals who are experts on CBT or are experts in other therapeutic approaches, General Practitioners, trained nurses, and non-professionals, such as women who had been diagnosed with PND themselves or who had experienced depressive episodes.

The targeted population of this review were 16 years or older. It was a requirement that they had either been diagnosed as suffering from PND and/or reported that they had been experiencing depressive symptomatology through self-report measures. Any studies with a population who were under the age of 16, or with a population that had been diagnosed or were suffering from other mental health or chronic health issues concurrently, in other words if they were mixed samples, were excluded. The mental health issues which were excluded were: personality disorders, developmental disorders, severe depression, anxiety, cognitive impairment, bipolar disorder, and psychotic disorders. The chronic physical health issues were diabetes, neurological disorders, stroke, physically handicapped, gastrointestinal problems, asthma, obesity, Alzheimer’s disease, Parkinson’s disease, and heart problems. Factors such as the socioeconomic background of the participants, educational level and/or marital status did not influence the selection of the targeted population.

For the purpose of this review, we included the following psychological interventions for the treatment of PND: CBT, cognitive therapy, psychoeducation, advice given, cognitive restructuring, behaviour management, goal setting, goal achieving, problem-solving therapy, mindfulness, stress management, relaxation, and breathing exercises.

A randomised controlled trial by Milgrom et al. [40] which we have also included in our Results Section, and which investigates the efficacy of CBT for PND through the internet, provides a comprehensive CBT model. It is called MumMoodBooster and it consists of six sessions. Each session focuses on different aspects of CBT and PND. The first session focuses on psychoeducation where information about PND and treatments are provided. The second session is about mood management and it talks about stress and anxiety, relaxation, and goals. The third session uses behaviour management where it explores issues such as life balance, goals, time management, and practicing change. The fourth session is about managing negative thoughts, while the fifth session focuses on increasing positive thoughts. The last session is about future planning where it explores the concepts of strategies, new routines, and commitment to change.

In addition the same program provides resources and has information on stress management, finding support, time management, and problem solving. It explores the concept of personal relationships with the focus on the person’s needs and also their partner’s. The program encourages the mother to meet the baby’s needs by “reading the cues” in the baby’s behaviour and to enhance the interaction between them through play. The basic need for sleep and strategies for improving it are also highlighted.

We excluded any studies from other schools of thought in psychology, such as the psychodynamic or humanistic approach, unless they were combined with other psychological approaches such as CBT, or in comparison to it for treating PND. The two main reasons for this choice were that CBT is “one of the best treatments which provides empirical evidence” [41], while psychodynamic or other psychotherapeutic therapies such as non-directive counselling are “unstructured and non-manualised” [42].

We included studies with control conditions that met the following criteria: typical primary care, waiting list, GP visits, clinic visits, home visits, anti-depressant medication, postnatal care, enhanced routine care with regular weekly or monthly visits by trained health workers, community treatment, referral to specialty services, and a single session focusing on debrief.

We conducted comparisons between various therapeutic approaches based on the following criteria:

1. The ratio of success of each treatment;
2. The duration of success of each treatment in terms of follow-ups. We included studies and follow-ups which varied in duration from one-week post-intervention to up to 5 years post-intervention;
3. The components of each treatment, e.g. what made each treatment successful.

We included studies in this review that used measures based on self-report questionnaires, such as the Edinburgh Postnatal Depression Scale [43], a valid and reliable scale that identifies the possibility of risk for the individual to develop perinatal depression [44], Hamilton Depression Rating Scale, Beck Depression Inventory, Global Assessment of Functioning Scale, Consumer Satisfaction Rating, Revised Clinical Interview Schedule, Therapist Rating Scale, Kruskal Wallis Test, Postpartum Adjustment Questionnaire, Social Adjustment Scale-Self-Report, and the Montgomery-Asberg Depression Rating Scale. We also included other studies which used formal diagnosis of PND based on clinical interviews of manuals such as the Structured Clinical Interview for DSM-III-R and DSM-IV.

We included studies which used measures such as depressive symptomatology, mood, coping strategies, social support, marital relationships, anxiety, social adjustment, relationship quality with partner, mother-infant relationship, suicidal ideation, suicide attempts, level of functioning, quality of life, health status, and sense of well-being.

There were no timing restrictions in terms of when studies were conducted. Studies included all types of settings. We reviewed studies published in the English language. Studies from research sources such as grey literature were not included.

We conducted a narrative review of the literature in four databases: Cochrane, PubMed, Scopus, and PsycINFO. The search took place on the 22nd and 23rd of December 2016. Reference lists of studies that were chosen initially from the four bibliographic databases were also reviewed and acted as secondary sources of information. Those reference lists were scanned, reviewed, and reported in detail accordingly. We also conducted another search in the same four bibliographical databases on the 23rd of December 2017. We wanted to find out whether there had been any published reviews or clinical trials for the treatment of PND from a psychological perspective in the year 2017.

We used 9 terms for our search: “postpartum depression” OR “treatment” OR “cognitive-behavioural therapy” OR “clinical trials” OR “randomised controlled trials” OR “reviews” OR “systematic reviews” OR “follow up”, AND “postpartum depression” OR “treatment” OR “virtual reality” OR “clinical trials” OR “reviews”. Our search took place in two parts. The first part focused on finding reviews and/or clinical studies on effective psychological evidence-based treatments for PND [45]. The second part focused on finding studies or reviews on VR as a treatment for PND.

We paid particular attention to clinical trials and randomised controlled trials, reviews and systematic reviews, CBT - VR treatment for PND. The search process and the inclusion and exclusion of reviews were cross checked by all authors independently. Any disagreements were resolved through consensus and with the support of an additional reviewer when necessary.

The selection process followed the following three steps:

1. Screened titles of studies to identify which could possibly fit the inclusion criteria;
2. Screened abstracts of the already chosen studies to further identify which better matched the inclusion criteria;
3. Screened the whole text in order to make sure that the studies chosen fit the inclusion criteria of our review.

If the authors identified any areas that needed clarification, they contacted the authors of those studies for ensuring those studies either fit the inclusion criteria or fit the exclusion criteria accurately. We kept a journal in which we recorded the reasons each study was included or excluded during the review process.

For the purpose of avoiding any risk of overlapping reports of the same study and to ensure avoiding bias and/or errors during the extraction data process, the extraction process was initially carried out by one reviewer. Data which focused on specific information, such as demographics, method, interventions, and outcomes were verified by the other reviewer(s) at a later stage. Any identified conflicts, misinterpretations, vague or grey areas were clarified by discussions between the reviewers and/or by contacting the authors of the studies selected, where necessary.

The results from our literature review search were recorded in an Excel spreadsheet with all relevant categories, such as studies, research design, intervention, and population, amongst others. The results were uploaded clearly and concisely based on the inclusion criteria and the keywords used for the search previously described.

We reduced bias and errors as all authors reviewed the studies separately and then later discussed any discrepancies identified.

They also identified the level of bias in terms of reporting. The authors divided the quality of each study into the following categories: yes, low, unclear, not strong, fair, and good. The decision for each of these categories for each study was based on the identification of reporting bias within the studies themselves. We considered issues in relation to selection bias, reporting bias, randomisation process, blinding of the participants, sample size, heterogeneity of methods used, generalisability of results, and limitations of each study.

We initially found 26 reviews in total. We also found 10 additional reviews through reference list searches, bringing the total of reviews up to 36. We examined all 36 reviews’ titles, names of authors, and year of publications and removed 14 reviews as duplicates. We examined the titles and abstracts of the remaining 22 reviews and we excluded 16 reviews as they did not meet the inclusion criteria of our review. We examined the remaining 6 reviews for eligibility and we included them in our review.

**Fig. 1:**
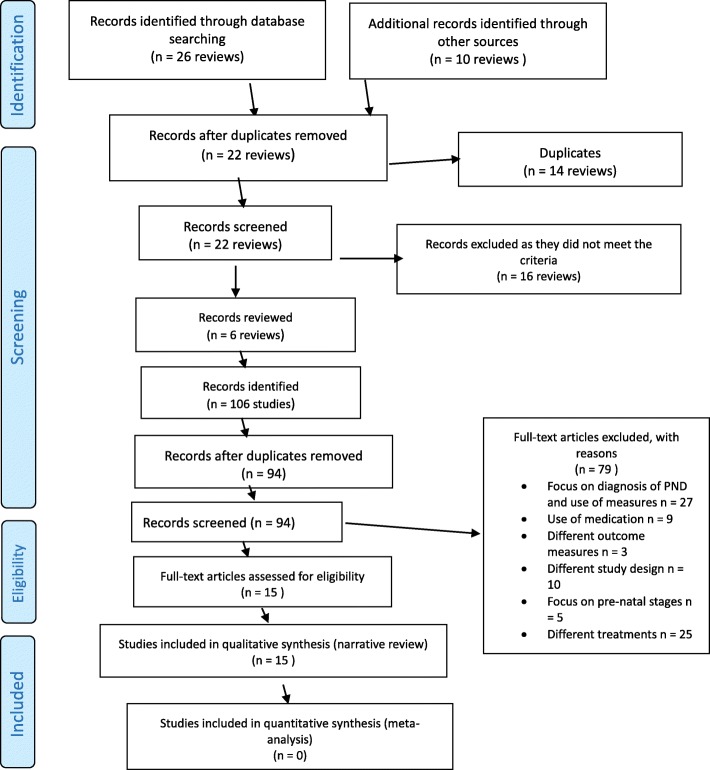
Flow chart of study selection process

We examined the 6 reviews that met our inclusion criteria and we found that they included 106 studies. We examined the names of the authors, and the year of publication, and we removed 12 of those studies as duplicates. We examined the title and abstract of the remaining 94 studies and we removed 79 as they did not meet our inclusion criteria. The final number of included studies was 15 (see Fig. 1 attached). Figure 1 is a flow chart which summarises the process of selection for the studies based on PRISMA template [46] which had been found up to December 2016. The authors of this review resolved any disagreement through discussion with further consultation from an additional reviewer, where necessary.

## Results

On our final list were 6 systematic reviews, one of which was a meta-analysis [47]. All six reviews included treatment studies with two reviews to include both prevention and treatment [47, 48]. We found no reviews or clinical trials published in the year 2017 that met our inclusion criteria.

All six reviews initially reviewed 1015 studies, of which 950 were excluded with the total of final studies included 106. The population of the six reviews was 24,231 in total. However review [48] did not provide the number of participants in the intervention group for two studies [49, 50], while in a second review [51], the number of participants in the intervention group was only reported in one out of the 10 included studies of that review.

There was a mix of pregnant and post-partum women, mothers, newly delivered mothers, and mothers and infants. Some participants had been screened for depression through a clinical interview, while others had reported depressive symptomatology through self-report measures.

The delivery of the interventions was a mixture of community based, including clinics and hospitals [47], home based [48, 51, 52], and a combination of individual and group therapy [47, 53]. One review [54] did not provide any information relating to the delivery intervention.

It was assumed that the number of interventions equalled the number of sessions, a total of 538.5. Some of the reviews provided information about the number of clinical hours used for the intervention [48, 53, 54], while one review did not provide any information relating to the number of clinical hours [51]. Some reviews reported missing information about the exact number of interventions in the studies they had reviewed [47, 51, 54].

The outcome measures were varied and included the Hamilton Depression Rating Scale, Beck Depression Inventory, with the most commonly used one being the Edinburgh Postnatal Depression Scale. There was an intention-to-treat analysis in four reviews [51–54].

There were a multitude of interventions reported in the six reviews, ranging from CBT, IPT, to psychodynamic, non-directive counselling, infant massage and others. The most frequently used intervention being CBT, followed by the IPT model.

The providers of the interventions were a mixture of professionals from various backgrounds, including psychologists, GPs, nurses and non-professionals such as lay women. There was no available information about the providers in two reviews [53, 54]. There is some missing data in terms of the duration of treatment and the number of sessions. We estimated the number of sessions to be approximately 610.5. There was a follow up assessment or intervention in 5 of the reviews, with only one exception [48].

In order to conduct a deeper analysis of the scientific literature, we applied our inclusion and exclusion criteria and extracted fifteen studies from the six reviews that met the inclusion criteria (see Table 1), 13 of them were randomised controlled trials, two studies were cluster randomised controlled trials [55, 56], and one was a randomised controlled trial with factorial design [50]. In the following sections we will describe the characteristics of the studies.

**Table 1:** Summary of the final list of the 15 studies for the treatment of PND and their characteristics

Name of author(s)	Setting – Location -dates	Target	Population	Delivery mode	Screening procedures	Outcome measures	Participants	Control group	Treatment group	Nature of Intervention	Intervention provider	Experimental conditions	Duration and number of sessions	Measure/ timescale	Follow up	Summary of main outcomes	Quality of study	Reporting bias	Limitations
[59] Ammerman et al. (2013 Adopted by [53]	USA	Treatment	Postpartum	Individual, home visits	EPDS, SCID, MDD diagnosis, HDRS, BDI-II, GAF, OTTF	SCID, HDRS, EPDS, BDI-II, GAF, OTTF, consumer satisfaction rating	93	46, control condition = home visits along with receiving treatment in the community	47	CBT related approach focused on stress management, goal setting, and problem solving	Clinicians	CBT vs routine primary care	15 h of contact	4.75, 7.75 months follow up	Yes	Overall positive outcome in favor of CBT versus typical care, “results found benefit at 4.5 and 7.5 months follow up”	Fair = good information on demographic population, same instruments administered in pre-treatment, post-treatment and follow ups,	Yes	“Population exhibited mild to moderate depression and the findings of this study cannot be generalised to the whole of population, e.g. severe depression, psychosis, etc., reporting bias, small study size, limited screening instruments, short term follow up, overall positive outcome in favor of CBT versus typical care but results were not statistically significant”
[49] Appleby et al. (1997) Adopted by [48, 51, 53]	South Manchester, England, May 1993-Feb 1995	Treatment	“Urban, community sample”, postpartum	Individual, home visits	EPDS, HAM-D, “Revised Clinical Interview Schedule”	“Revised Clinical Interview Schedule”-EPDS, HDRS	87	Not applicable as all four study groups received some kind of intervention	87	Placebo and 6 CBT sessions fortnightly - CBT focused on techniques such as challenging and modifying negative automatic thoughts and “dysfunctional beliefs”, increasing pleasant stimuli and reducing behaviours which could have a negative effect on mood	Psychologist with no previous clinical experience	Medication (Fluoxetine) and 1 CBT session, medication and 6 CBT sessions, Placebo and 1 CBT session, Placebo and 6 CBT sessions	“6 biweekly CBT sessions”	1, 4, and 12 weeks post-treatment	Yes	“Immediately post-intervention, all 4 groups showed significant improvement on the Revised Clinical Interview Schedule, Fluoxetine, an anxiolytic antidepressant, is an effective treatment for PND, A course of six sessions of CBT is more effective than a single session, there seems to be no advantage in receiving both medication and counselling at the same time, the simplest treatment after a single session of CBT may be fluoxetine as it removes the need for additional counselling, Many women with PND are reluctant to take medication”	“Fair as clinical interview was used but results of CBT effect is unclear as the use of medication is used in one of the groups, however they used independent assessors to evaluate study outcomes, not clear about allocation concealment”	Unclear	“30% attrition rates, exclusion of participants with chronic depression, combination of medication and CBT as the results were less successful in comparison to the use of medication itself”
[58] Bennett (2001) Adopted by [51]	United Kingdom	Treatment	Women identified for “probable depression”	Group	EPDS	EPDS	45	22, control condition = standard primary care with health visitor	23	Not manualised CBT, psychoeducation, relaxation techniques	Health visitors	8 weekly two hour sessions	Immediate and 24 weeks post-treatment	Yes	CBT has a good therapeutic effect on post-natal depression	Not strong as there was small sample, limited use of instruments, “unclear if caregivers were blinded”	Unclear	“Small sample size”, it is demanding in terms of commitment on behalf of the participants, it is expensive and time consuming, high percentage of participants failing to “complete” treatment,	
[50] Chabrol et al. (2002) Adopted y [48]	Toulouse, and Narbonne, France	Prevention/ Treatment	Women identified with depressive symptoms	Individual, home visits, clinic visits	BDI, EPDS, HAM-D	BDI, EPDS, HAM-D	48	30, control condition = routine care /clinic visits	18	“CBT with elements of psychoeducation, supportive and psychodynamic approach”, CBT focused on techniques such as challenging and modifying negative automatic thoughts and dysfunctional beliefs, increasing pleasant stimuli and reducing behaviours which could have a negative effect on mood	“Master’s Degree level therapists”	5–8 home visits /6 weekly one hour sessions	“Immediately post-intervention”	No	“Immediately post-intervention, women in the intervention group had reduced scores on all measures compared to women in the control group”	Not strong as small sample, lack of follow up, no reporting of size of control and experimental groups in the review, however they “used manualised interventions and attempted to ensure adherence to the treatment protocol”	Yes	“Non-independent outcome assessment, small sample size, lack of follow-up”	
[60] Cooper et al. (2003) Adopted by [48, 53]	Participants homes, hospital settings-Cambridge, England, Jan 1990- Aug 1992	Treatment	Women with post-partum depression, being primiparous, living close to maternity hospital, and English as their first language	Individual, home visits	EPDS	Therapist Rating Scale, Kruskal-Wallis test, EPDS, DSM-III-R (SCID)	193	52, control conditions: routine primary care provided by the general practitioner and health visitors	Counselling = 48, CBT = 42, Psychodynamic = 48	“Psychodynamic, non-directive counselling, and CBT which focused on issues in relation to the infant that the mothers would find difficult to cope with, and on issues in the mother’s relationship with the infant, mothers were given support through advice to manage difficulties, used problem-solving techniques, challenged thinking patterns”	6 in total where 3 experts in each one of the three treatments, and three non-specialists health visitors	“Routine primary care, non-directive counselling, CBT, psychodynamic therapy”	10 weekly sessions of either CBT, psychodynamic, or non-directive counselling	4.5 months, 9 months, 18 months, 5 years post-partum	Yes	“All three interventions had better clinical outcomes than the control group, the psychodynamic group had a superior clinical effect in comparison to the other two treatments and to the control group on depression at 4.5 months post-partum, this changed subsequently at 9 months, 18 months and five years, where no real difference between control and intervention groups were reported”	Good as they “ensured adherence to the clinical protocol”	Yes	“Not all specialists were familiar with home visiting but only the health visitors, thus some of the therapeutic outcomes in the groups of participants run by specialists, CBT and non-directive counselling were very low”
[62] Honey et al. (2002) Adopted by [53, 54]	United Kingdom	Treatment	“Newly delivered mothers”	Individual, home visits	EPDS	EPDS	45	22, control conditions = routine primary care administered by health visitors	23	“PEG, education, coping strategies, CBT techniques which focused on stress management, goal setting, and problem solving”	Not reported	CBT home visits vs routine care	8 × 2 hour weekly sessions	8 months follow up	Yes	Significant reduction in depression between intervention group and routine primary care	Fair as they used a single self-report measure, short-term follow up, a combination of interventions used	Yes	“Population exhibited mild to moderate depression and the findings of this study cannot be generalised to the whole of population, e.g. severe depression, psychosis, etc., not enough information about population demographics except their age and mean, reporting bias, small sample size, small study size, limited screening instruments (only EPDS), short term follow up, overall positive outcome in favor of CBT versus typical care but results were not statistically significant, not clear what is the clinical effect of CBT as there is a mix of interventions”.
[63] Milgrom et al. (2005) Adopted by [53, 54]	Australia	Treatment	“Newly delivered mothers-Postpartum”	Group, individual, home visits	BDI	BDI	192	46, control conditions = care provided by health nurses	C1 = 47, C2 = 66, C3 = 33	“C1 = CBT (coping with depression course), C2 = CBT-related,C3 = Group-based Cognitive-Behavioural Therapy including psychoeducation, role-playing, discussion, stress management, goal setting, and problem solving”	Not reported	Usual care	9 × 90 minute weekly sessions	3 months follow up	Yes	“Significant depression score reductions in all interventions in comparison to routine primary care”	“Fair due to lack of generalisibility of results, small study size, lack of information, but adequate number of sessions and good information on the interventions themselves”	Yes	“Population exhibited mild to moderate depression and the findings of this study cannot be generalised to the whole of population, e.g. severe depression, psychosis, etc., not enough information about population demographics except their age and mean, reporting bias, small study size, limited screening instruments (only BDI), overall positive outcome in favor of CBT versus typical care but results were not statistically significant”
[65] Milgrom et al. (2011) Adopted by [53]	Australia	Treatment	Postpartum	Individual, public hospital, home visit, GP practice	EPDS, BDI-II	BDI-II	68	Not applicable	GP management = 23, counselling + CBT delivered by nurse-22, counselling + CBT delivered by psychologist = 23	“CBT approach focused on stress management, goal setting, and problem solving”	GPs, primary nurses, psychologists	Management by trained GP vs. Counselling-CBT delivered by a trained nurses vs. Counselling-CBT delivered by a psychologist	3 h of contact	2 months follow up	Yes	All three interventions were effective for treating PND	Not strong due to lack of control group, short term follow up, a mix of intervention providers were used	Yes	The size of sample was small, attrition rates were relatively high, no real control group, reports of medical practitioners instead of standardised interviews were used, single psychologist vs. multiple nurses, no long-term follow up, low referral ratio and treatment uptake, “results were not statistically significant”
[61] Misri (2004) Adopted by [51]	Canada	Treatment	Postpartum women having been diagnosed with depression	Individual	HRSD, EPDS	HRSD, EPDS	35	16, typical care = antidepressant medication in a hospital outpatient program	19	“CBT which was based on a treatment manual focused on challenging and modifying dysfunctional beliefs, and correcting the information processing of the individuals”	Psychologist	Weekly 1-h CBT sessions plus antidepressant medication vs standard care (antidepressant medication)	12 one hour sessions plus medication	12 weeks post-treatment	Yes	CBT has a good therapeutic effect on PND	Not strong as “it provided data on anxiety”, “the timing of the final outcome assessment was immediately post-treatment”, “blinding of caregivers was not possible as they were involved in the intervention”	Yes	Small sample size, it is demanding in terms of commitment on behalf of the participants, it is expensive and time consuming, high percentage of participants failing to “complete” treatment, CBT is combined with medication, no reporting of size of control and experimental groups, however they “used manualised interventions and attempted to ensure adherence to the treatment protocol”
[55] Morrell (2006) Adopted by [51]	United Kingdom	Treatment	Postpartum women identifying with depressive symptomatology through self-report measures	Individual, home visits	EPDS	EPDS	595	191, control conditions = participants referred to general practitioners by health visitors	404	“CBT treatment focused on modifying dysfunctional beliefs, and correcting the information processing of the individuals”	“Health visitors, nurses”	A weekly basis for one hour up to a maximum of 8 weeks, CBT, and non-directive counselling vs. standard primary care	8 one-hour sessions	24, 52, and 72 weeks postpartum	Yes	“It compared psychological with psychosocial interventions” CBT has a good therapeutic effect on post-natal depression, non-directive counselling can also be effective in treating post-natal depressive symptomatology	Not strong as big sample size but no information on the number of participants on control and experimental groups in the review, use of self-report measures	Not clear	It is demanding in terms of commitment on behalf of the participants, it is expensive and time consuming, high percentage of participants failing to “complete” treatment, high attrition rate at 24 weeks post-partum, no information on the number of participants on the control group and intervention group
[16] O’Hara (2000) Adopted by [51, 52]	United States	Treatment	Women identified through a multi-stage community screening process for depression, “social adjustment, marital relations, and postpartum adjustment”	Individual	SCID, HRSD, BDI, PAQ, SASSR	HRSD, BDI, PAQ, SASSR	120	51, control conditions = waiting list	48	Interpersonal psychotherapy using psychosocial and psychological components compared to a waiting list	“Trained therapists”	Interpersonal psychotherapy vs waiting list	12 h sessions over 12 weeks	“4, 8, and 12 weeks post-randomisation”	No	“IPT is an efficacious treatment for postpartum depression. It reduced depressive symptoms and improved social adjustment, and represents an alternative to pharmacotherapy, particularly for women who are breastfeeding”, improvement on all questionnaires	Fair as the sample size is descent, population is diagnosed with major depression, use of multiple instruments, use of trained therapists, but no follow up, positive results on mother-infant relationships do not reflect the relationship with the newborn baby	Not clear	No follow up so long-term effect of treatment is unknown, doesn’t measure the relationship between mother and newborn baby, not clear if the intervention was delivered at home or was clinically based
[57] Prendergast and Austin (2001) Adopted by [48, 51, 53]	Australia	Treatment	Women identified with depression-“Community sample”-postpartum	Individual	DSM-IV, EPDS	EPDS, MADRS	37	20, control conditions = “standard care with 6 weekly clinic visits lasting 20 to 60 min”	17	Home visits-“CBT sessions”, CBT focused on techniques such as challenging and modifying negative automatic thoughts and dysfunctional beliefs, increasing pleasant stimuli and reducing behaviours which could have a negative effect on mood	“Early Childhood Nurses”	CBT vs standard care (“weekly clinic visits”)	“6 weekly CBT sessions”	10 weeks post-partum	Yes	No difference between the two groups post-intervention but better outcome for the intervention group six months follow up but not statistical significant	Fair as “they followed participants over time” “used manualised interventions and attempted to ensure adherence to the treatment protocol”	Not clear	“Although the efficacy of the interventions has been demonstrated for some outcomes, effectiveness studies are required to establish whether such benefits would be obtained in routine practice”, 55% drop out rate for control group, small sample size, no “intention-to-treat analysis”
[56] Rahman (2008) Adopted by [47]	Pakistan	Treatment	Pregnant women, who were married, between 16 and 45 years old, and had depression	Individual	DSM-IV clinical interview, HDRS	DSM-IV clinical interview, HDRS	903 (105 lost in follow up)	440 (54 lost in follow up), control conditions = routine care with regular weekly visits in the last month before birth, and monthly visits after that by health workers	463 (51 lost in follow up)	Home based CBT intervention which was part of a community health program called “Thinking Healthy”, CBT approach used pictures and structured activities for achieving specific everyday goals	“Community health workers”	“Enhanced care involving home visits” vs. usual care	16 sessions in 11 months, 1.5 sessions per month	6 and 12 months postnatally	Yes	“Non-mental health professional can deliver positive psychosocial interventions with good therapeutic outcomes in middle-income countries”	Fair as this study examined antenatal and post-natal depression and the timing of the intervention was delivered in two stages, in the third trimester of pregnancy and ten months postnatally, adequate info on attrition rates of population in the “final analysis”, “information on follow up was adequate”	Yes, “low risk of bias on blinding of participants and personnel, and of outcome assessment, and no selective reporting”	“Highlights stigma of depression on mothers and unrealistic to expect mothers would be supported to participate in studies as such, resource demanding in resource limited countries due to the use of professionals over a lengthy period of time, disadvantaged over preventive treatments, also disadvantaged due to individual delivery vs. Group based”, “unable to carry out a subgroup analysis of treatment versus preventive interventions because only one treatment intervention was identified”, “CBT was part of psychosocial management of post-natal depression and included elements such as psychosocial improvement, helping the individual to consider a general sense of wellbeing”
[64] Rojas (2007) Adopted by [54]	Chile	Treatment	Newly delivered and low income mothers	Groups	EPDS	EPDS	230	116, usual care = GP consult with antidepressant medication, and referral to specialty services if needed	114	CBT focused on psychoeducation	Trained doctors, midwives, nurses	Group CBT vs. usual care	8 × 50 minute weekly sessions	3 month and six month follow up	Yes	“Mothers with newborn babies and on low income can benefit from multi-component medication, bigger improvement in three months than six months”	Fair, good sample size but only use of self-report measure, not very long follow up,	Yes	Unclear around the “purity” of CBT intervention (“multi-component intervention”), combination of CBT with psychoeducation and pharmacology, EPDS is not a diagnostic tool, unclear of the reason(s) the initial therapeutic effect of three months to six months post-intervention was reduced
[66] Wiklund et al. (2010) Adopted by [53]	Sweden	Treatment	Postpartum - mothers with signs of depression	Individual, maternity clinic	EPDS	EPDS	67	34, standard care = a single session with a midwife or obstetrician focusing on debrief	33	CBT approach focused on “stress management, goal setting, and problem solving”	Midwives	CBT vs standard care	21 h of contact	2.75 months follow up	Yes	“Brief CBT is effective in treating women with signs of depression”	Fair as small sample size and use of self-report measure, sufficient time of intervention allocation, short-term follow up	Yes	“Population had not been diagnosed with post-natal depression, and thus findings of this study cannot be generalised, not enough information about population demographics, reporting bias, small study size, limited screening instruments (only EPDS), short term follow up, results not statistically significant, not certain on long-term effectiveness of CBT, lack of clinical diagnostic procedure”

*all sentences in “quotes” are an exact copy of the original statement from the authors, and all abbreviations are included after the limitations section

### Quality of studies

The quality of the studies varied from not strong to very good, with most to be considered fair.

This was based on the randomisation process, sample size, heterogeneity of methods, use of instruments, treatment protocol, generalisability and statistical significance of results, follow ups, and limitations of each study. Most studies reported bias except five studies for which it was unclear [16, 49, 55, 57, 58].

### Treatment focus

Fourteen studies focused solely on the treatment of PND, one on the treatment of ante-natal depression and PND [56], and one on prevention and treatment of PND [50].

Almost all studies, except one [16], focused mostly on depressive symptomatology of the mother as a primary outcome measure.

### Population studied

In the 15 studies the population, which in total were 2758, were either diagnosed with depression or had identified themselves as depressed. More specifically in six of the 15 studies the population were post-partum women who had been diagnosed through a clinical interview based on the DSM-IV [16, 49, 57, 59–61]. In one of them, the population were 16 years and older [56]. In the remaining 9 studies the participants would mostly identify with depressive symptomatology, mostly through interview based questionnaires such as HAM-D, or self-report questionnaires such as EPDS. In 2 out of the 9 studies the populations were “newly delivered mothers” [62, 63], and in one study, they were newly delivered mothers with low income [64].

### Control groups

The control groups were made up of participants who would usually receive typical primary care, or they were on a waiting list. However, two out of the fifteen studies in the review did not have a control group [49, 65]. For example, the study by Appleby et al. [49] included four study groups which all received some kind of intervention. The study by Milgrom et al. [65] included three groups which all had some type of intervention.

In addition, it was not clear what the control conditions were for two other studies [58, 62]. For example, in the study by Honey et al. [62] the control conditions were routine primary care administered by health visitors, and in the study by Bennet et al. [58] the control conditions were standard primary care with a health visitor. However it was not clear whether, in either studies, the routine primary care involved GP visits, medication, both, or none.

In the remaining 11 studies, the control conditions were as follows: waiting list [16], health visitors contacting participants, and defining their postnatal care with the use of questionnaires and referring them to their general practitioners [55], enhanced routine care with regular weekly visits in the last month before birth, 1 month post birth and monthly visits for the next 9 months by routinely trained health workers who received regular supervision but they were not specialised in CBT [56], routine care in the form of clinic visits [50], home visits which focused on “child health and development, nurturing mother-child relationship, maternal health and self-sufficiency”, along with receiving treatment in the community [59], routine primary care which “would be typically provided by the primary health care team such as the general practitioner and health visitors with no additional input from the research team” [60], antidepressant medication received by control group subjects in a hospital outpatient program [61], “standard care with 6 weekly clinic visits lasting 20 to 60 minutes” [57], health nurses who would manage case by case the participants and refer them to other services where appropriate [63], antidepressant medication, brief psychotherapeutic interventions, GP consult, or referral to specialty services [64], and a single session with a midwife or obstetrician focusing on debrief [66].

### Delivery of the interventions

In terms of the delivery of the interventions, 12 were individual-based and home visits, 2 were group-based [58, 64], and 1 study was carried out on an individual basis and was also group based [63]. Most interventions were delivered in the homes of the participants. One study was delivered at home and in a public hospital. One study provides no data relating to delivery of the intervention.

### Location of the studies

Five studies took place in the United Kingdom, three studies in Australia, two studies in the United States, one study in France, one study in Canada, one in Pakistan, one study in Chile, and one study in Sweden.

### Number of sessions and content of the interventions

The interventions in all of the studies varied in terms of the number of clinical hours and number of sessions. The majority of studies provided the number of sessions and number of clinical hours. However, 4 of the 15 studies [49, 56, 57, 60] provided only the number of sessions and not the number of clinical hours. The total number of clinical hours was approximately 168.5, with 1648 people having been provided with at least one of those interventions. The average number of clinical hours for each participant was 9.78. The average treatment period was 12.1 weeks.

The interventions were CBT based and most studies were a comparison between CBT and usual primary care. However one study compared 4 groups which all received some kind of treatment. The experimental conditions were medication with 1 CBT session, medication with 6 CBT sessions, placebo with 1 CBT session, and placebo with 6 CBT sessions [49]. Another study also did not have a control group but rather three intervention groups [65]. 2 CBT interventions included elements of psychoeducation, cognitive restructuring, and relaxation exercises [58, 63], one study compared CBT delivered at home vs CBT delivered in a clinic [50], one study compared the three main interventions, i.e. CBT, non-directive counselling, and psychodynamic, and in comparison with usual care [60], one study used CBT and the psychodynamic approach [5], one intervention compared CBT delivered either by psychologists, nurses, and GPs [65], another study compared CBT in combination with medication vs. primary care [61], and lastly one study compared interpersonal psychotherapy vs. a waiting list [16]. In Table 2 we included a summary of the components included in the intervention protocols and the number of studies that used each of the components.

**Table 2:** Summary of all CBT components for the treatment of PND

Treatment	Number of studies
Psychoeducation (i.e., Advice given for supporting mothers to manage difficulties, mother-infant relationship issues)	9
Cognitive restructuring (i.e. Challenging and modifying negative automatic thoughts and dysfunctional beliefs, information processing correction)	9
Problem solving	7
Behaviour management: Increasing pleasant stimuli or reducing behaviours which could have a negative effect on mood	6
Goal setting and Goal achieving daily using pictures and structured activities	6
Stress management	6
Relaxation	1

### Intervention providers

The intervention providers varied from nurses, psychologists, GPs, health visitors, and midwives. Almost all, except two studies [16, 50], included follow-ups varying from 1 week post-partum to 5 years following the birth of the child. The average period of follow-ups was 6.14 months.

### Clinical trials using VR for the treatment of PND

We found no clinical trials that used VR for the treatment of PND. However, we found three studies in total [40, 67, 68] which used some form of technology. Two of them [67, 68] used video recordings, mostly for supervision purposes. The third study [40] was internet based for the delivery of CBT.

## Discussion

### What CBT psychological treatments are effective for PND?

All fifteen studies included in our review used CBT as the main treatment for PND. However, there were 9 studies which compared CBT to other treatments such as non-directive counselling, psychodynamic and primary care, and 3 studies where CBT was combined with non-directive counselling, psychodynamic and primary care.

It appears that CBT can be viewed as a large clinical territory with many different techniques for the treatment of the same mental health issue. For example, in one study, CBT emphasised psychoeducation [64] while in other studies CBT focused on challenging negative thoughts and dysfunctional beliefs [55, 57]. In another study CBT was part of a wider community based program [64]. In the study by Rojas, the “purity” of the CBT approach was questionable [64].

We measured 7 components of CBT that were used for the treatment of PND, which were psychoeducation, cognitive restructuring, problem-solving, behaviour management, goal setting and goal achieving, stress management, and relaxation (Table 2).

The two most frequent used CBT interventions were found to be psychoeducation and challenging negative thoughts and beliefs with 9 studies in total having employed both at different times.

The second most frequent CBT intervention that was used was problem solving, while the third most frequently used CBT interventions were goal setting, behaviour management, and stress management.

### What are the parameters that make those treatments having a successful clinical outcome?

This review shows the general outcome is that CBT as a therapeutic intervention is effective for the treatment of PND. It has an advantage over primary care for reducing depressive symptomatology in the post-partum period. However, the severity of PND varied in the included studies in our review, with most of the participants to be in the mild to moderate range. Thus it is unknown whether the same positive clinical results of CBT would be obtained for the more severe range of depression.

Another issue that was identified in relation to the effectiveness of CBT was the significance of the results. In five out of fifteen studies [60, 62, 63, 65, 66] it was found that their results on CBT’s effectiveness were positive overall but not statistically significant.

It is important to mention that although our review focused on CBT there were studies that compared CBT with other psychotherapeutic approaches that also showed effectiveness. It was found that non-directive counselling and psychodynamic approaches also had a positive effect on the reduction of symptoms of PND. To be more precise in the study conducted by Cooper et al. [60] which examined CBT, non-directive counselling and psychodynamic therapy, and compared them with typical primary care, it was found that all three interventions were effective. CBT was more effective on cognitive focus, behavioural tasks, and organisation. Nonetheless, the psychodynamic approach was more effective on relationships. It was also superior to the other two interventions in terms of depressive symptomatology according to structured interviews, especially up until the fifth month post-intervention. However, past that point, the initial therapeutic effect of all three interventions had started diminishing. From 9 months onwards up until 5 years post-intervention, the initial therapeutic effect was virtually non-existent.

Other studies have found similar results, whether the outcome is measured by self-reported measures or by a mental health professional conducting a clinical interview. CBT can be an effective treatment for PND in the short-term but its clinical effect long-term is questionable [65]. The same study measured the combination of CBT with counselling delivered either by a psychologist or a nurse. They found that the two approaches and a third one which was GP management, mainly through medication, had a good overall effect in the treatment of PND. We can conclude that CBT is an effective approach that can be delivered by various mental health professionals of different backgrounds, or even delivered by non-experts, such as lay people, or health visitors [56, 58].

It can also be concluded that CBT can be delivered in a flexible manner, whether through a home visit, or in a public place such as a hospital or clinic [65, 66]. There does, however, appear to be a preference toward home visits as it is believed to be more convenient and accessible to post-partum women with depression.

In addition to the treatment type and location CBT can be delivered in a brief manner which can have good therapeutic outcomes. According to Wiklund et al. [66] there is some evidence that brief CBT can benefit PND in the mild to moderate range. However it is worth noting that the population of that study were women who had not been diagnosed with depression but who only experienced signs of depression.

From the studies of this review we can conclude that CBT can also be delivered on an individual level or in a group [58, 63]. It seems that the individual delivery of the intervention is preferential and maybe the potential of the group intervention of CBT has not been adequately investigated. We can safely assume though that group intervention might have some advantages over individual treatment such as utilising less resources. At the same time it might be a disadvantage in terms of participants who might be more reluctant to participate initially in a group setting, where its participants share the same psychological experiences. This possibly underlines stigma on a societal level but this needs to be further investigated.

The studies of this review also show that CBT’s positive therapeutic outcome on PND is not impacted by the socioeconomic status of the population. Post-partum women with depression can benefit equally whether they are from a lower-middle income country or high income country. This review includes studies from different countries and continents and are categorised differently according to the GDP per capita, e.g. Australia vs. Chile [64, 65].

Another conclusion of this review is around the outcome measures. Most studies in this review measured individual depressive symptomatology in the post-partum period. However, one study [16] focused on the mother’s depression and measured it as a primary outcome. The authors included additional information, e.g. mother-infant relationship. It is worth mentioning here that the relationship between mother-infant was not in relation to the newborn baby, but rather on the already existing children in the family.

An issue that was raised was in relation to the target disorder and the self-reported measures which were used by the participants of some of the studies. The self-reported measures indicate depressive methodology but they do not necessarily ensure a formal diagnosis of depression. There was a lack of clinical diagnostic protocols in some of the studies.

A difficulty that one of the studies highlights was in relation to CBT. CBT was considered to be time consuming and highly demanding in terms of resource intervention [55]. Treating PND with populations of low socioeconomic characteristics, or in countries of low to middle income would prove to be a challenging task. Providing treatment to depressed mothers overall has been proven to be a challenging task on its own, especially in relation to the delivery of intervention. It becomes clear that home visits are a preferable way of delivery over GP practice or a public hospital for varying reasons. A young mother, or a mother with a high-demanding household might find it difficult to transport herself outside her home for a number of reasons, including a lack of transportation, money or time.

Another issue that was highlighted in our research was around the stigma of mental health issues. One study highlights the difficulties a clinical population, or mental health professionals might encounter in rural areas or low-income countries [56]. One way that the authors were able to deal with this issue was to support the idea that the CBT intervention was part of a larger community health program. Another way was that they promoted the idea of the infant’s health and well-being as a priority.

Some studies had a high attrition rate up to 30% but not all of them. It was reported that some participants did not complete the treatment. There was also a variation in terms of their length, and number of phases in the follow up process. All studies except two [16, 50] had follow ups with variations in frequency the follow ups were conducted for the majority of the studies and the span of the time period over which this happened, e.g. 2 months versus 5 years.

Almost all studies included different criteria in relation to population, e.g. primiparous vs. pregnant women vs. post-partum women, with different socioeconomic backgrounds and from different countries. We also did not have enough or adequate demographic information for all the included studies, which would allow us further analysis and conclusion reaching in our review process.

### Has VR been used in the treatment of PND?

From our investigation, we found no clinical trials that had used VR as a form of treatment for PND. However, we found three studies which used technological means in clinical practice. One study investigated the efficacy of Toddler-Parent Psychotherapy (TPP), for the improvement of parents’ and infants’ mental health [67]. The technological means that were employed during this study were videotapes which had recorded the interaction between the mother and the infant. However, the videotapes were used for supervision purposes to ensure the “fidelity of the intervention” itself.

Another study investigated whether CBT could be delivered through the internet [40]. They used a specific program called MumMoodBooster. However, this did not include any VR elements.

A third study used video recordings to assess the interaction between mothers and infants [68]. The intervention used in the study was infant massage in a support group.

We have identified the lack of clinical trials using VR for the treatment of PND as a gap in the literature. For this reason we are planning to conduct two clinical studies following this review. The first one will be a pilot study which will measure the feasibility of using VR for the treatment of PND. This will act as a prerequisite which will guide us to conduct a clinical trial where we will investigate what effect, if any, VR has on traditional therapies for PND.

### Limitations

As a narrative review, this study has some limitations. It is missing some of the qualities and advantages of the methodologies that a systematic review and/or a meta-analysis can provide. This review does not give strong emphasis on the methodological flaws of the initial studies included in it. It also does not provide any statistical analysis of the data of the included studies which could highlight issues around variations in individual studies, heterogeneity, or effect size. In addition this review does not assess risk of bias in a systematic way. It does not use any specific tool to assess bias in regards to allocation concealment, blinding of participants, incomplete outcome data, and selective reporting. This review reports on the quality of studies and the reporting of bias within the studies but rather in a brief and not in-depth way.

Another limitation was around the strict inclusion/exclusion criteria we used, and as a result, the total number of studies included was limited. We excluded studies of different type, such as pilot studies, observational studies, and case studies. We also excluded grey literature such as unpublished data, which otherwise might have offered a different or richer perspective.

We excluded studies which had a different target disorder. In most cases, we included studies only for the treatment of PND. We did not touch upon the prevention of PND, prevention or treatment of ante-natal depression, management of ante-natal or post-natal anxiety or distress.

We decided not to include any studies which did not measure as a primary outcome, the depressive symptomatology of the participants. For example we excluded studies which had different outcome measures such as mother-infant relationship. Although, it is known that the mother’s emotional state can affect the infant in different aspects of their lives at a later stage, such as their cognitive or emotional development. However, for the purpose of this review, we decided to exclude any studies that did not have the depressive symptomatology of the participants as the main or primary outcome measure, which is limiting in itself.

## Conclusions

Taking into consideration the limitations of a narrative review, we believe our study contributes to the literature on various levels.

We were able to identify reviews in the literature which had examined various treatments for PND. These varied from psychosocial and psychological interventions, to health promotion, massage, and exercise. However, we wanted to focus on CBT treatments for PND. For this reason, we searched for clinical trials which had used CBT as their main treatment. We were able to extract data on what CBT treatments have been used. We identified 7 main CBT components frequently used for the treatment of PND.

Researching mainly CBT treatments for the treatment of PND is one of the main contributions of this review to the current literature as we offer a rich CBT perspective for the treatment of PND. In other words we subtracted only the studies from the reviews that met our inclusion criteria in relation to types of interventions. For example, review [53] included 18 studies but only 8 of them met our inclusion criteria as that review investigated not only treatment but also screening of depression ante-natal and post-natal population. We borrowed only 6 out of 10 studies in another review [51] as it investigated a broader range of treatments for PND. Review [49] provided us with 4 suitable to our criteria studies out of 6 studies in total due to its focus on treatment but also prevention of PND, while review [54] with 3 out of 7 studies due to different study designs. Lastly, two reviews [47, 52] provided us with only one study each, with the first review having included 10 studies, and the second one with 8 included studies. The reasons were due to different types of interventions and different outcome measurements respectively. In addition, only two studies [48, 56] had been used by three different reviews at the same time [48, 51, 53].

Another important contribution of this review was the identification of CBT’s parameters and what makes it an efficacious clinical approach for PND. We identified parameters such as the delivery of CBT, the providers, and what measures were used, amongst others. An interesting finding was that intervention providers come from wide and varied backgrounds, both professionals and lay women.

The multitude of CBT parameters also depicts a richness of therapeutic approaches within the CBT spectrum, which highlights flexibility. CBT can be delivered by various providers, and in different ways. It is a highly effective clinical approach, but it has its clinical limitations. For example, CBT’s efficacy is time limited in the sense that it is effective for up to 6 months. Its impact plateaus after that time.

We also found a gap in the literature indicating there have been no studies using VR for the treatment of PND. This leads us to the question of whether VR could be used as a possible treatment intervention method of PND. The next step will be to test this intervention. We aim to run a pilot study and measure its feasibility as a preparation for conducting a clinical trial at a later stage.

## Abbreviations

12-I-GHQ: 12 Item General Health Questionnaire
ASSP: Ainsworth Strange Situation Procedure
BDI: Beck Depression Inventory
BDI-II: Beck Depression Inventory
BSID: Bayley Scales of Infant Development
BSQ: Behavioural Screening Questionnaire
CBT: Cognitive-behavioural therapy
CES-D: Centre for Epidemiologic Studies Depression Scale
DMC: Dyadic Mutuality Code
DSM-5: Diagnostic and Statistical Manual of Mental Disorders – Fifth Edition
DSM-III-R (SCID): Diagnostic and Statistical Manual of Mental Disorders – III – R
DSM-IV: Diagnostic and Statistical Manual of Mental Disorders – Fourth Edition
EPDS: Edinburgh Postnatal Depression Scale
ESDS: Epidemiological Studies Depression Scale
GAF: Global Assessment of Functioning Scale
GHQ: General Health Questionnaire
GSPI: Goldberg’s Standardised Psychiatric Interview
HAM-D: Hamilton Depression Rating Scale
HDRS: Hamilton Depression Rating Scale
HIV: Human Immunodeficiency Virus
HRSD: Hamilton Rating Scale for Depression
IPT: Interpersonal Psychotherapy
K-10-IS: Kessler 10-Item Scale
MABI: Mother’s Assessment of the Behaviour of the Infant
MADRS: Montgomery-Asberg Depression Rating Scale
MDD: Major Depressive disorder
MDIBSID: Mental Development Index of the Bayley Scales of Infant Development
MSCA: McCarthy Scales of Children’s Abilities
NBAS: Neonatal Behavioural Assessment Scale
OTTF: Outside Treatment Tracking Form
PAQ: Postpartum Adjustment Questionnaire
PBCL: Pre-school Behaviour Checklist
PCERA: Parent-Child Early Relational Assessment
PCMD: Perinatal Common Mental Disorders
PEG: Controlled Psychoeducational Group
PHQ: Patient Heath Questionnaire
PND: Post-Natal Depression
PPAQ: Postpartum Adjustment Questionnaire
PPD: Post-Partum Depression
PSI: Parenting Stress Index
RCT: Randomised Controlled Trials
SAS: Social Adjustment Scale
SASSR: Social Adjustment Scale-Self-Report
SCID: Structured Clinical Interview for DSM-IV
SRQ: Self-Reporting Questionnaire
WPPSI: Wechsler Preschool and Primary Scales of Intelligence
